# Novel Browning Agents, Mechanisms, and Therapeutic Potentials of Brown Adipose Tissue

**DOI:** 10.1155/2016/2365609

**Published:** 2016-12-25

**Authors:** Umesh D. Wankhade, Michael Shen, Hariom Yadav, Keshari M. Thakali

**Affiliations:** ^1^Arkansas Children's Nutrition Center, Little Rock, AR, USA; ^2^Department of Pediatrics, University of Arkansas for Medical Sciences, Little Rock, AR, USA; ^3^Duke University, Durham, NC, USA; ^4^Diabetes, Endocrinology, and Obesity Branch, National Institutes of Health, Bethesda, MD, USA

## Abstract

Nonshivering thermogenesis is the process of biological heat production in mammals and is primarily mediated by brown adipose tissue (BAT). Through ubiquitous expression of uncoupling protein 1 (Ucp1) on the mitochondrial inner membrane, BAT displays uncoupling of fuel combustion and ATP production in order to dissipate energy as heat. Because of its crucial role in regulating energy homeostasis, ongoing exploration of BAT has emphasized its therapeutic potential in addressing the global epidemics of obesity and diabetes. The recent appreciation that adult humans possess functional BAT strengthens this prospect. Furthermore, it has been identified that there are both classical brown adipocytes residing in dedicated BAT depots and “beige” adipocytes residing in white adipose tissue depots that can acquire BAT-like characteristics in response to environmental cues. This review aims to provide a brief overview of BAT research and summarize recent findings concerning the physiological, cellular, and developmental characteristics of brown adipocytes. In addition, some key genetic, molecular, and pharmacologic targets of BAT/Beige cells that have been reported to have therapeutic potential to combat obesity will be discussed.

## 1. Introduction

Thermogenesis is the process of biological heat generation that plays a crucial role in the homeostatic regulation of body temperature in warm-blooded animals. The primary mode of nonshivering thermogenesis in mammals is via the metabolic activity of brown adipose tissue (BAT). BAT ubiquitously expresses the mitochondrial transmembrane protein thermogenin, otherwise known as uncoupling protein 1 (Ucp1) [1]. Ucp1 uncouples oxidative phosphorylation from ATP synthesis by increasing the permeability of the inner mitochondrial matrix and allowing leakage of protons. This mitochondrial proton leak reduces the efficiency of the cellular respiratory machinery, resulting in an increase in heat energy dissipation [2]. Because of its vital role in modulating an organism's global energy expenditure, BAT has been the subject of great interest for its therapeutic potential in the treatment of diabetes and obesity.

**Figure 1:**
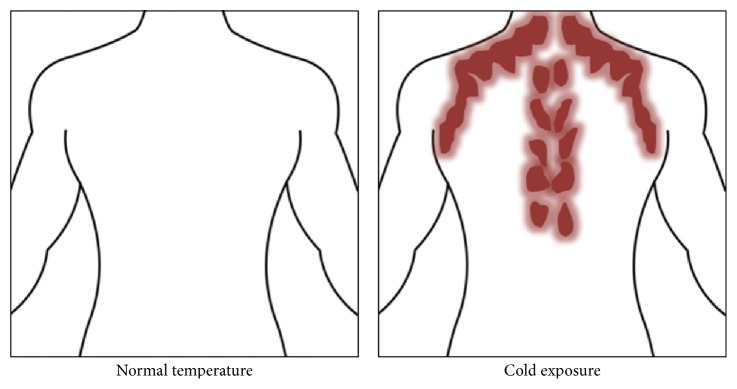
Location of brown adipose tissue activity as detected by PET-CT with 18F-FDG. BAT is highlighted in red and gets activated upon cold exposure (figure remade and adopted from van Marken Lichtenbelt et al. [5]).

BAT has been canonically associated with small mammals such as rodents and in newborns and infants of larger mammals such as humans. However, a recent profusion of studies in functional imaging and molecular biology has demonstrated that BAT is a functionally active tissue in adult humans [3–5]. BAT is found primarily in the supraclavicular and neck region and along the spine in humans and interscapular region in rodents. Thus, BAT is generally thought to be limited to distinct local depots, while white adipose tissue (WAT) is more broadly distributed throughout the body. The primary cell type within BAT is multilocular brown adipocytes, which exert thermogenic effects through the expression of Ucp1 in the inner mitochondrial membrane [6]. Because of its putative evolutionary role in protecting against hypothermia, it was traditionally believed that BAT was only transiently present in infants since thermoregulation is most necessary during the early phases of life. However, over the last decade, metabolic imaging and radiological studies based on the use of isotopic glucose analog fluorodeoxyglucose (FDG) have provided conclusive evidence that BAT exists in adult humans. In fact, some early FDG-PET/CT (positron emission tomography/computed tomography) scans noted “tumor-like” substrate uptake in the regions suggested to contain BAT [5, 7, 8] (Figure 1). The mean quantity and relative distribution of BAT are significantly decreased by adulthood in humans, but it has been purported that even a small quantity of BAT can have substantial metabolic effects.

**Figure 2:**
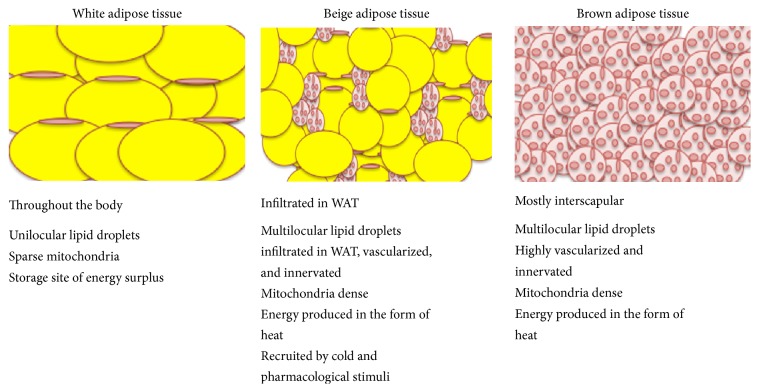
Cartoon representation of different types of adipose tissue depots.

WAT, highlighted by large unilocular adipocytes, is the primary site of surplus energy storage and has additionally been regarded as a critical endocrine organ (Figure 2). WAT releases hormones (leptin, adiponectin, etc.) and cytokines (tumor necrosis factor-*α* (TNF-*α*), interleukin 6 (Il-6), monocyte chemoattractant protein-1 (Mcp-1), etc.) that modulate whole-body metabolism, insulin resistance, and the systemic low-grade inflammation associated with obesity [9, 10]. Additionally, WAT plays a role as a thermal insulator and also acts as a shock absorber when it comes to protecting other organs from mechanical damage [11]. In addition to the canonical definitions of white and brown adipocytes, recent research has demonstrated a population of cells that can exhibit BAT-like characteristics in response to certain external cues but that can reside primarily in WAT. These have been dubbed “beige” or “brite” (brown in white) adipocytes and are capable of expressing Ucp1 and exhibiting thermogenic capacity comparable to classical brown cells in response to various environmental and pharmacological stimuli [12] (Figure 2). In particular, the induction of beige adipocytes has been demonstrated to occur under exercise and cold exposure and in response to *β*-adrenergic agonists such as CL 316,243. Similar to adipocytes in BAT, beige cells in mouse WAT are defined by their multilocular lipid droplet morphology, high mitochondrial content, and the expression of a core set of brown fat specific genes such as Ucp1, Cidea, and peroxisome proliferator-activated receptor-gamma coactivator (Pgc1*α*).

Both brown adipocytes and beige cells have the potential to be metabolically beneficial due to their unique thermogenic property, which has the potential to alter the balance between energy intake and energy expenditure. As mentioned earlier, nonshivering thermogenesis is the process of uncoupling protons from ATP synthesis, and the excess energy is dissipated as heat. The canonical pathway of nonshivering thermogenesis begins with sympathetic activation and norepinephrine release to activate *β*_3_ adrenergic receptors. This in turn increases cAMP production to activate protein kinase A (PKA), which leads to increased activity of several intracellular lipases and decreased perilipin activity. The resulting net lipolysis leads to release of free fatty acids (FFAs) which are taken up into mitochondria via carnitine palmitoyltransferase 1 (CPT1) in the outer mitochondrial membrane. Once, inside the mitochondria, FFAs remove purine inhibition of Ucp1, causing influx of H^+^ protons into the mitochondrial matrix, uncoupling oxidative phosphorylation and energy from the proton motive force is dissipated as heat. Many of the genes related to glucose uptake and catabolism are upregulated in BAT from cold-exposed mice [13]. In obese, glucose-intolerant mice, cold exposure can normalize glucose tolerance and increase both glucose and fatty acid uptake in BAT of lean and obese mice [14, 15]. BAT can also take up glucose in an insulin-independent matter. Teperino et al. demonstrated that there is robust insulin-independent glucose uptake in BAT and skeletal muscle via activation of the Smoothened- (Smo-) AMP-activated protein kinase (AMPK) axis [16], indicating that energy sensing by AMPK in BAT may regulate fuel utilization. Fatty acids secreted from adipose tissue are esterified to become available for *β*-oxidation or reassembly for storage as inert TG via lipogenesis. In the BAT of cold-exposed rodents, genes involved in glucose metabolism, lipogenesis, and uptake and catabolism of fatty acids are upregulated as part of cold adaptation and fatty acids are utilized for UCP1 activation [13]. PPAR*γ* in the endothelium of BAT and vascular endothelial growth factor B (VEGF-B) in heart and skeletal muscle control the uptake of FAs to regulate and integrate vascular and metabolic responses adverse circumstances [17–19].

The activity of these cells has been negatively associated with diet-induced obesity in animal models, while ablation or loss of function has resulted in increased susceptibility to diet-induced obesity. The last decade has witnessed numerous studies elucidating the physiology of BAT and beige cells, as well as potential small chemical, gene, and molecular targets to increase their activity. Since several excellent reviews have examined BAT biology in the last few years [20–22], the present review does not intend to provide an exhaustive view of the field. Instead, we will examine the developmental origins of brown and beige cells, transgenic models of browning, and environmental and chemical agonists of browning in adipose tissue. For example, the role of the central nervous system in control of BAT function and thermogenesis has been extensively and excellently reviewed elsewhere [23–25]; thus this will not be reviewed here. This review will focus on recently published literature from the past two years (2014–2016) regarding browning of adipose tissue. Finally, we will provide perspective on the current therapeutic potential of BAT/Beige cells to combat obesity.

## 2. Origins of Brown and Beige Cells

BAT is one of the earliest fat depots to form during embryonic development and is assumed to contain a uniform population of adipocytes, which express Ucp1. The major BAT depots in rodents are in the interscapular region (interscapular, axillary, and cervical pads) embedded in and around deep back muscles. Thus it is plausible that brown adipocyte precursors exist in these depots to replenish this pool in response to developmental or environmental cues. In human infants, interscapular BAT is the most prominent depot and plays a critical role in maintaining infant body temperature during the first years of life. Interscapular BAT in human infants is known to regress with age and is almost absent in adults [26, 27]. Most brown fat cells originate from precursor cells in the embryonic mesoderm that also give rise to skeletal muscle cells and a subpopulation of white adipocytes [28, 29]. Pluripotent mesenchymal stem cells are capable of developing into both brown and white adipocytes, with Pgc1*α* and Ppar-*γ* being important factors in each differentiation process, respectively. Considering the developmental relationship between brown fat and muscle, it is not surprising that brown fat precursor cells express a gene signature very similar to premyocytes prior to differentiation [30]. Furthermore, brown fat and muscle share mitochondrial proteomes [31]. Seale et al. demonstrated through lineage tracing of adipocytes in vivo that depots of BAT but not WAT arise from* myf5*-expressing precursors, a myogenic regulatory factor that regulates skeletal muscle myogenesis [29, 32]. However, the lineage specificity of brown and skeletal muscle progenitor cells is still under investigation and further lineage tracing experiments need to be done to conclusively designate these cells as progenitors for a particular population.

The developmental origin of beige or inducible brown adipocytes resident in WAT depots is an important and understudied question. Current work supports the hypothesis that brown adipocytes resident in WAT depots are of a completely distinct developmental lineage than brown adipocytes in classical BAT depots. This led to the acceptance of inducible brown adipocytes as a new cell population and subsequent naming of these cells as beige cells [29]. This notion has been supported by work with primary cultures derived from gonadal WAT, which demonstrated a thermogenic gene program expressed in response to PPAR*γ* agonists that was distinct from that of cultures of classical brown fat cells from the interscapular depot [33]. With regard to the origins of these recruited brown adipocytes, transdifferentiation during adipogenesis and de novo synthesis have been posited as two possibilities. Using a lineage tracing approach, Wang et al. showed that most of the newly emerging beige adipocytes that appear in subcutaneous depots are not derived from existing white adipocytes. In fact, there may be a dedicated set of beige precursor cells that undergo beige adipocyte differentiation, as opposed to the transdifferentiation of existing adipocytes [34]. However, the transdifferentiation hypothesis is not completely understood, although the effects may be a depot-dependent. In particular, several groups have reported that beige adipocytes found in abdominal WAT are derived de novo, whereas the vast majority of beige adipocytes in inguinal fat involve conversion of existing white adipocytes to a beige phenotype, as previously proposed [35–37].

## 3. Transgenic Models of Browning 

### 3.1. Gain of Function Transgenic Models of Browning

Certain genes have been established to play important roles in the development of BAT and the induction of beige cells. Some of the identified markers of BAT include Ucp1, PR domain containing 16 (Prdm16), forkhead box C2 (FoxC2), and cyclooxygenase 2 (Cox2), and overexpressing these genes increases beige adipocyte induction and improves mitochondrial bioenergetics and overall BAT function. For instance, agouti viable yellow (A^vy^) genetically obese mice demonstrate reductions in total body weight and subcutaneous fat stores when WAT-specific Ucp1 was overexpressed. Analysis of gonadal fat revealed an increase in a novel adipocyte type that did not accumulate lipids and that constituted approximately 80% of the mass of the WAT tissue of the A^vy^ transgenic mice [38]. Another study using the same approach showed that apolipoprotein-2 (Ap2) driven adipose tissue specific overexpression of Ucp1 made mice resistant to obesity induced by a HFD, presumably due to ectopic synthesis of Ucp1 in WAT. These animals exhibited atrophy of BAT, as indicated by reduced brown fat mass and reduction of systemic Ucp1 and mitochondrial DNA content [39].

Prdm16 is another brown/beige specific marker that has been shown to be critical for development of brown-like adipocytes in subcutaneous, but not gonadal, adipose depots. Prdm16 overexpressing transgenic mice display increased energy expenditure, limited weight gain, and improved glucose tolerance in response to a HFD. In addition, Prdm16 haploinsufficiency reduces browning in WAT when stimulated by *β*-adrenergic agonists. These results demonstrate that Prdm16 is a cell-autonomous determinant of the BAT gene program and thermogenesis in subcutaneous adipose tissues [40].

Cox2 is a rate-limiting enzyme in prostaglandin synthesis. Previously, others have shown that Cox2 plays a critical role in whole-body energy homeostasis and adipose tissue metabolism; for example, selective inhibition of Cox2 was shown to attenuate weight loss and energy expenditure in cancer patients and tumor-bearing mice [41, 42], and genetically manipulated mice that express only one wild-type allele of Cox2 were shown to exhibit fat accumulation [43]. Vegiopoulos et al. showed that overexpression of Cox2 in WAT induced de novo brown adipocytes recruitment, increased systemic energy expenditure, and protected against HFD-induced obesity. Cox2 is a downstream effector of *β*-adrenergic signaling in WAT and is required for the induction of BAT in WAT depots [44, 45]. Another interesting target to consider is lipoxin A_4_ (LXA_4_), which like Cox2 is also involved in arachidonic acid metabolism. Adipose tissue from transgenic mice overexpressing arachidonate 5-lipoxygenase activating protein (Alox5ap), an important mediator in leukotriene formation, had higher LXA_4_ than leukotriene B_4_ levels, and these mice were leaner and showed increased energy expenditure in part due to the browning of WAT. Moreover, upregulation of hepatic liver x receptor (LXR) and Cyp7a1 led to increased bile acid synthesis, which may have contributed to increased thermogenesis due to the correlation between bile acids and Ucp1 activity [46]. In addition, transgenic Alox5ap mice were protected against diet-induced obesity, insulin resistance, and inflammation, with browning of WAT being the prominent mechanistic finding from this study [47].

Some genes that have been highlighted in cancer, such as Foxc2, folliculin, and Pten, may also be involved in browning pathways. Overexpression of Foxc2 expression in adipocytes has a pleiotropic effect on gene expression, which leads to a lean, insulin sensitive phenotype. Foxc2 affects adipocyte metabolism by increasing the sensitivity of the *β*-adrenergic-cAMP-PKA signaling pathway through alteration of adipocyte PKA holoenzyme composition to ultimately induce a beige adipocyte phenotype [48]. Also, overexpression of Foxc2 in HFD-fed mice was associated with reduced fat mass and complete protection from diet-induced insulin resistance, intramuscular accumulation of lipid, and hepatic insulin resistance [49]. Pten is a known tumor suppressor in several forms of cancers and overexpression of Pten results in increased energy expenditure and protection from metabolic pathologies in mice. Further analysis revealed that BAT in Pten-overexpressing mice is hyperactive and these mice present with high levels of Ucp1, which is reported to be a target of Foxo1 [50]. Folliculin (FLCN), known for its role as a tumor suppressor, may play a crucial role in metabolic reprogramming of adipose tissue and shift to an oxidative phenotype. Adipose tissue specific deletion of FLCN via an adiponectin- driven cre led to increased energy expenditure and protection from HFD-induced obesity. Mitochondrial Ucp1 and other markers of brown fat are upregulated in both white and brown FLCN-null adipose tissues, explaining the increased resistance of Adipoq-FLCN knockout mice to cold exposure. Mechanistically, FLCN ablation leads to chronic hyperactivation of AMPK, which in turn induces and activates three key transcriptional regulators of cellular metabolism, PPAR*γ*, Pgc1*α*, and ERR*α*. The AMPK/Pgc1*α*/ERR*α* molecular axis positively regulates the expression of key metabolic genes to promote mitochondrial biogenesis and activity [51].

Adrenomedullin 2 (AM2) is a secreted peptide belonging to the calcitonin gene-related peptide (CGRP) superfamily and plays an important role in regulating cardiovascular homeostasis. Interestingly, it has been found that AM2 protein expression is significantly decreased in the adipose tissue of obese mice. When these obese mice were treated with exogenous AM2, blood glucose, fasting serum insulin, and free fatty acid levels were all significantly reduced whereas glucose tolerance and insulin sensitivity were improved. Follow-up work with aP2/AM2 transgenic mice that had aP2 promoter driven AM2 expression in adipose tissue under HFD conditions corroborated these effects. In particular, reduced weight gain, improved glucose tolerance, and insulin sensitization were observed. Importantly, the aAM2-tg mice displayed increased energy expenditure. These effects may be due to increased AMP-activated protein kinase phosphorylation and reduced Pgc1*α* acetylation, which would result in favorable interactions between Pgc1*α*, Prdm16, and Ucp1 in adipocytes [52].

### 3.2. Loss of Function Transgenic Models of Browning

A number of transgenic studies have demonstrated that loss of function of certain crucial genes and proteins is known to diminish the browning potential of adipose tissue. For instance, Bone Morphogenetic Proteins (BMPs) have been shown to play a role in brown adipogenesis and designating the commitment of cell fate to a brown phenotype. In fact, a number of studies corroborated that members of the BMP superfamily are involved with adipocyte lineage and differentiation [53–55]. BMP7 knockout mice have a marked paucity of interscapular brown fat at birth, suggesting that BMP7 plays critical role in embryonic BAT development. Interestingly, a significant role in browning of WAT has been observed for transforming growth factor beta (TGF-*β*), a close relative of the BMP family. TGF-*β*/Smad3 signaling downregulation confers protection against diet-induced obesity and associated comorbidities. Smad3-deficient mice gain significantly less weight when fed a HFD and display significantly increased mitochondrial biogenesis. The metabolic improvements in Smad3 knockout mice were attributable to the increased induction of the BAT phenotype in WAT [56].

Another protein involved in browning that has been explored through loss of function studies is serine hydrolyzing enzyme, *α*/*β* hydrolase domain containing 6 (*α*/*β*HD6). *α*/*β*HD6 can act as a monoacylglycerol hydrolase and is believed to play a role in endocannabinoid signaling as well as in the pathogenesis of obesity and liver steatosis [57]. Loss of ABHD6 in HFD-fed mice resulted in modestly reduced food intake, decreased body weight gain, improved glucose tolerance and insulin sensitivity, and enhanced locomotor activity. Knockdown of *α*/*β*HD6 led to increased energy expenditure, cold-induced thermogenesis, Ucp1 expression in WAT, fatty acid oxidation, and browning of WAT. Furthermore, when the *α*/*β*HD6 inhibitor WWL70 was given to WT mice, the effects of adipose browning and cold-induced thermogenesis were replicated, suggesting that *α*/*β*HD6 is an important mediator of adipose browning [58].

Members of the NF-*κ*B signaling pathway, known for its role in inflammation and insulin signaling, may also provide interesting insights into BAT regulation. In one study, deletion of immediate early response gene X-1 (IEX-1), a downstream target of NF-*κ*B, protected mice against HFD-induced adipose and hepatic inflammation, hepatic steatosis, and insulin resistance. Deletion of IEX-1 in mice led to markedly less weight gain on chronic HFD feeding and these affects were attributable to increased energy expenditure. IEX-1 deficiency induced browning and activated the thermogenic gene program in WAT but not in BAT by promoting alternative activation of adipose macrophages. Consequently, IEX-1^−/−^ mice exhibited enhanced thermogenesis, providing a plausible explanation for the increased energy expenditure and lean phenotype observed in these mice [59].

Liver X Receptors (LXR) were shown to repress expression of Ucp1 in classical BAT depots in one rodent study. In particular, LXR*β* suppressed the induction of the BAT phenotype in subcutaneous WAT in male rodents fed a normal diet. Depletion of LXRs activates release of thyroid-stimulating hormone (TSH) from the pituitary which, after a number of signal transduction events, is thought to lead to the browning of WAT [60]. The secretory product C-terminal fragment of Slit2, a member of the Slit extracellular protein family, acts via Prdm16 in order to regulate beige adipocyte induction. Slit2-C fragment is a cleaved product that has an active thermogenic properties, promoting adipose thermogenesis, augmenting energy expenditure, and improving glucose homeostasis in vivo. Mechanistically, Slit2 induces robust activation of PKA signaling, which is required for its prothermogenic activity [61]. Mice with mTORC1 impairment, through either adipocyte-specific deletion of* RAPTOR *or pharmacologic rapamycin treatment, were refractory to the well-known *β*AR-dependent increase of Ucp1 expression and expansion of beige adipocytes in WAT. PKA directly phosphorylates mTOR and RAPTOR on unique serine residues. Abrogation of the PKA site within RAPTOR disrupts *β*AR/mTORC1 activation of S6K1 without affecting mTORC1 activation by insulin [62].

### 3.3. MicroRNAs and Browning

MicroRNAs are emerging in a wide variety of fields as key regulators of development and disease. MicroRNAs are small (~22 nucleotides) noncoding RNAs found in plants, animals, and some viruses that play a role in posttranscriptional regulation of gene expression via RNA silencing [63]. With regard to BAT, miR-455 is a potential microRNA target which has been demonstrated to play a role in brown adipogenesis. miR-455 exhibits a BAT-specific expression pattern and can be induced by cold exposure and the browning regulator BMP7. Moreover, adipose-specific miR-455 transgenic mice display marked acquisition of brown fat (BAT) characteristics in subcutaneous WAT upon cold exposure. The effects of miR-455 are thought to be mediated by activation of AMPK*α*1 through targeting of hypoxia inducible factor 1 *α* subunit inhibitor (HIF1an) as AMPK promotes the brown adipogenic program and mitochondrial biogenesis. Concomitantly, miR-455 also targets the adipogenic suppressors Runx1t1 and Necdin, initiating adipogenic differentiation [64].

Exosomes are small (40–100 nm in diameter), membrane-bound vesicles that are released from several cell types after fusion with the plasma member. The main molecules found inside exosomes are lipids and proteins, but several types of nucleic acids, including miRNAs, are also found in exosomes. Thus, when released, exosomal miRNAs can affect the function of recipient cells. BAT activation enhances exosome release from BAT. Profiling the miRNAs in exosomes released from brown adipocytes and in exosomes isolated from mouse serum revealed that levels of miRNAs change after BAT activation both in vitro and in vivo. In particular, serum concentrations of exosomal miR-92a, a common miRNAs present in both rodent and human serum exosomes, are inversely correlated with human BAT activity as measured by ^18^F-FDG-PET/CT in a cohort of 41 healthy individuals. This work suggests that exosomal miR-92a could be used as a potential serum biomarker for BAT activity in mice and humans in the future [65].

To summarize, our understanding of the browning process has been furthered by identifying key molecules via gain or loss of function approach; however, elucidation of mechanistic pathways will help to develop clinical aspects of browning research. The browning process varies with each adipose tissue depot in question as does expression of the key molecules mentioned above. In addition, it is yet to be seen how well findings from rodent studies recapitulate in humans. Nonetheless, the use of transgenic rodent models of browning has considerably advanced our understanding of the role of browning in obesity.

## 4. Pharmacological/Chemical Agents and Additional Pathways Involved in Browning 

Advances in the area of BAT research have led to the emergence of several pharmacological and plant-based browning agents that stimulate brown adipogenesis or beige cell induction under certain conditions. In a pioneering study, Himms-Hagen et al. showed that chronic treatment with CL 316243, a *β*3 adrenergic receptor- (AR-) selective agonist, increased body temperature and 24-h energy expenditure, mainly by increasing resting metabolic rate. Interscapular BAT showed three- to fourfold increases in Ucp1 and cytochrome oxidase content. In addition, some multilocular adipocytes appeared in normally almost exclusive unilocular WAT depots (e.g., mesenteric, inguinal, epididymal, and retroperitoneal depots). The authors from this study conclusively demonstrated that CL 316243 not only promotes BAT mitochondrial proliferation and thermogenesis, overall energy expenditure, and leanness but also retards the development of WAT hyperplasia during the early stages of diet-induced obesity [66]. Further studies by Guerra et al. demonstrated that treatment with CL 316243 increased levels of Ucp1 mRNA in retroperitoneal fat of male A/J mice to levels comparable to those found in interscapular brown fat. This increase in Ucp1 correlated with weight loss in response to treatment with CL 316243 [67]. Recent studies have demonstrated that CL 316243 also stimulates beige cell formation in typical WAT depots. Granneman et al. showed that standard *β*3 AR stimulation by CL 316243 for 7 days led to the development of beige cells in WAT [68]. The same group subsequently reported that a 75% reduction in CL 316243 dose was still effective, resulting in diminished fatty acid-induced inflammation and greater proliferation of inducible BAT progenitors in abdominal WAT [69]. Thus, in rodents, *β*3AR activation with CL 316243 is an established and potent method of BAT phenotypic induction.

Several other pharmaceuticals have been reported to have off-target effects related to browning. For example, Gleevec, a tyrosine kinase inhibitor and antineoplastic agent mainly known for its role in treating chronic myeloid leukemia, improved insulin sensitivity when administered to HFD-fed mice. Furthermore, Gleevec reduced lipogenic and gluconeogenic gene expression in the liver and ameliorated inflammation in adipose tissues. Finally, Gleevec increased browning of WAT and the rate of energy expenditure, putatively by blocking PPAR*γ* phosphorylation [70].

Administration of the short chain fatty acid acetate in nanoparticle form to HFD-fed mice resulted in increased heat production from brown or beige adipocytes. Increased thermogenic output in the acetate-treated animals was also corroborated with significantly increased Ucp1 expression. Prdm16, another classical marker of the brown/beige adipocyte, was significantly increased in the adipose tissue of acetate-treated animals. In addition, the key transcriptional coregulator, Pgc1*α*, similarly showed a trend toward increasing. Mitochondrial modulations in adipose tissue may also explain how acetate reduces whole-body adiposity without appetite suppression [71].

**Figure 3:**
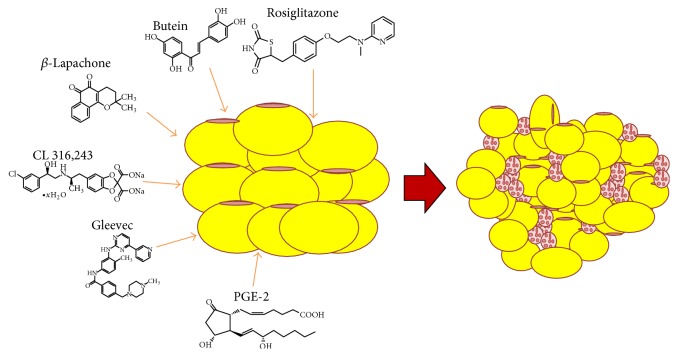
Chemical/plant by products that induce browning in white adipose tissue.

Thiazolidinediones (TZDs), known PPAR agonists used for glycemia management in diabetes, may induce a phenotypic transition of WAT toward brown fat. One study examined peptide-functionalized nanoparticles containing the TZD rosiglitazone or a prostaglandin E2 analog (16,16-dimethyl PGE2). When injected into adipose tissue vasculature of mice, rosiglitazone promoted both transformation of WAT into brown-like adipose tissue and angiogenesis. Intravenous administration of these nanoparticles can target WAT vasculature to stimulate angiogenesis that is required for the transformation of adipose tissue and transform WAT toward a brown phenotype via the upregulation of angiogenesis and BAT-specific markers. Moreover, in a diet-induced obese mouse model, these angiogenesis-targeted nanoparticles were shown to inhibit body weight gain and modulate several serological markers including cholesterol, triglyceride, and insulin [72] (Figure 3). Liraglutide, another drug prescribed for weight loss, is known to improve type 2 diabetes in humans and works through activation of the glucagon-like peptide 1 receptor (GLP-1R) in the brain. When liraglutide was centrally administered to mice, it stimulated BAT thermogenesis, adipocyte browning, and suppression of food intake with subsequent weight loss. In obese type 2 diabetic patients, exenatide and liraglutide (both GLP-1R agonists) treatment for one year led to increased energy expenditure [78].

Certain phenols and plant derivatives have also been suggested to possess adipocyte modulatory functions. For example, Ucp1 can be induced by the small molecule butein, a plant polyphenol that mediates its action through induction of Prdm4. This pathway ultimately leads to increased energy expenditure and stimulates the generation of thermogenic adipocytes [74]. Resveratrol, a natural phenol and phytoalexin produced by several plants, displays similar effects to butein when given to mice. Resveratrol reduced adipose tissue inflammation, as indicated by the decreased expression of the proinflammatory cytokines Tnf*α* and IL-6 in adipose tissue. Concomitantly, there was increased expression of genes associated with the browning of adipose tissue, including Ucp1 and Pgc1*α* [75]. *β*-Lapachone (BLC) is a naturally occurring quinone plant compound that has been implicated in browning. BLC stimulated the browning of WAT, increased the expression of BAT-specific genes such as Ucp1, decreased body weight gain, and improved metabolic parameters in HFD-fed mice. BLC-treated mice showed significantly higher energy expenditure compared to control mice. At the cellular level, BLC increased the expression of brown adipocyte-specific genes in stromal vascular fraction- (SVC-) differentiated adipocytes. BLC mediated its effects via controlling the expression of miR-382, which led to the upregulation of its direct target, Dio2. Upregulation of miR-382 markedly inhibited the differentiation of adipocytes into beige adipocytes, whereas BLC recovered beige adipocyte differentiation and increased the expression of Dio2 and Ucp1 [76] (Figure 3). Capsaicin, another plant-based compound, has been found to stimulate the expression of brown adipocyte-specific thermogenic Ucp1 and Bmp8b in WAT. Capsaicin triggered browning of WAT by promoting sirtuin-1 (SirT1) expression and activity via TRPV1 channel-dependent elevation of intracellular Ca^2+^ and phosphorylation of Ca^2+^/calmodulin-activated protein kinase II and AMP-activated kinase. Capsaicin increased the expression of PPAR*γ* 1 coactivator *α* and enhanced metabolic and ambulatory activity. Further, capsaicin stimulated SirT1-dependent deacetylation of PPAR*γ* and Prdm16 and facilitated PPAR*γ*–Prdm16 interaction to induce browning of WAT. However, administration of dietary capsaicin did not protect TRPV1^−/−^ mice from obesity [77] (Table 1). Importantly, in humans, a single oral dose of capsinoids, less-pungent capsaicin-like compounds, increased energy expenditure selectively in people with metabolically active BAT, demonstrating that capsaicin and related capsinoids stimulate thermogenesis in both humans and rodents [79].

Exercise, while not a pharmaceutical, is another commonly used intervention in the treatment of obesity and obesity-associated cardiometabolic complications. In 2012, Boström's group showed that the exercise-induced, muscle derived myokine, Irisin plays a critical role in browning of adipose tissue. Irisin acts on WAT in culture and in vivo through activation of Pgc1*α* and stimulates Ucp1 expression and wide brown fat-like development [80]. Meteorin-like protein (Metrnl) is a circulating factor that is also induced in muscle after exercise and in adipose tissue upon cold exposure. Increased circulating levels of Metrnl stimulate energy expenditure, improve glucose tolerance, and increase the expression of genes associated with beige fat thermogenesis and anti-inflammatory cytokines. Blocking Metrnl in vivo significantly attenuates chronic cold-exposure-induced alternative macrophage activation and thermogenic gene responses [81]. Knudsen et al. in an independent study showed that Il-6 is required for exercised training-induced increase in inguinal WAT Ucp1 mRNA content. Daily injection of Il-6 for a week also was able to increase Ucp1 mRNA content in inguinal WAT. Cold exposure-induced increase in UCP1 protein content observed in WT mice is markedly reduced in IL-6 KO mice suggesting the requirement of Il-6 [82].

In another exercise related study, Morton et al. fed female C57BL/6 mice either a control or HF diet for 6 or 18 weeks and subjected them to an exercise or sedentary regimen. Interestingly, quadricep triglyceride levels were significantly increased in both exercised and sedentary mice fed HFD for 18 weeks. In addition, Ucp1 and Pgc1*α* increased with exercise in HF diet fed mice. Increased Ucp1 and Pgc1*α* in the exercised myocytes of running mice suggest that a beige/brown fat phenotype may have developed. Findings from this study suggest that increased muscle lipid may develop a “brown” phenotype in the setting of endurance exercise training, a shift that is further promoted by HFD [83]. However, the main limitation of this study is the inability to distinguish between intramyocellular and extramyocellular lipid; thus whether increased lipid levels and “browning” occur in myocytes or in myoadipocytes remains to be determined.

## 5. Microbiome and Browning

A symbiotic relationship between host metabolism and gut microbiome and its role in metabolic health and diseases is well documented [84]. The gut microbiota contributes to host metabolism through dynamic changes in metabolites, nutrients, and vitamins and contributes to maintenance of normal energy homeostasis [85]. As the largest endocrine organ, the gastrointestinal tract also secretes various regulatory peptide hormones that control several physiological processes including whole-body energy metabolism [86]. Impairment of symbiotic equilibrium between the host and gut microbiota contributes to the pathophysiology of obesity [87]. Since the microbiome plays a critical role in energy homeostasis and is a probable source of key signaling molecules, it is possible that the microbiome plays a role in BAT biology or browning of adipose tissue. For example, Chevalier et al. demonstrated that gut microbiome remodeling during cold exposure plays an important role in browning of adipose tissue [88]. Cold exposure dramatically shifted microbial composition into the gut and was tightly correlated with induction of browning activity. Interestingly, microbiome transplantation from cold-exposed mice to germ free mice significantly increased insulin sensitivity in recipient mice and enhanced cold tolerance via increasing BAT activity. These observations strongly suggest that alterations in the gut microbiome are a key factor that modulates energy uptake by increasing absorption area in intestine and modulating whole-body energy metabolism during increased energy demand [88]. Similarly, Suárez-Zamorano et al. [89] showed that depletion of the gut microbiome helps to increase browning of WAT and ameliorate obesity and associated defects in leptin deficient and diet-induced obese mice. In this study, microbiome depletion enhanced M2 macrophage signaling (anti-inflammatory macrophages) in adipose tissue that helped to populate beige cells in WAT, resulting in improved insulin sensitivity. In contrast, blocking M2 macrophage signaling or recolonization of the microbiome deteriorated browning in WAT with increased insulin resistance [89]. These results demonstrate that the gut microbiome regulates beige fat development to alter the host response to metabolic adversity.

Sexually dimorphic and strain-dependent differences in metabolic responses to dietary challenges are well documented [90, 91]. It has been well documented that C57Bl/6J male germ free mice exhibit lower body fat mass than their conventional counterparts and are resistant to HFD-induced obesity [92]. On the other hand, C57Bl/6J conventional male mice (with an intact microbiome) gain weight faster than female counterparts, suggesting that differences in body weight gain are dependent on gender [92, 93]. In 2012, Mestdagh et al. demonstrated that the gut microbiome contributes to body weight gain in a gender-dependent manner in mice and does so by regulating BAT physiology [93], since gender-dependent differences in the development of diet-induced obesity were associated with changes in gut microbiome and BAT fat oxidation activity. Moreover, microbiome depletion induced elevation of specific metabolites, that is, 3-hydroxybutyrate and lactate, in BAT, suggesting that the microbiome contributes to regulation of fat and glucose metabolism in BAT.

The physiological metabolic function of the gut requires metabolites such as short chain fatty acids (SCFAs) produced via fermentation of dietary fibers by anaerobic intestinal microbiota [94]. SCFAs are an integral metabolite for normal gut function, are known to modulate host energy metabolism, and contribute to the pathophysiology of obesity and diabetes [95]. While the role of SCFAs in regulation of host metabolism and their underlying mechanism(s) are still the subject of rigorous research, microbiome-derived SCFAs influence metabolic function of various peripheral energy-sensitive organs such as the liver, skeletal muscle, brain, pancreas, and adipose tissue (WAT and BAT). Gao et al. [73] reported that SCFAs induced increased expression of Ucp1 and Pgc1*α* in BAT, thereby increasing thermogenesis and fatty acid oxidation and providing protection against HFD-induced obesity in mice. In another study consistent with findings reported above, SCFAs produced in response to butyrate-producing probiotics (VSL#3) significantly reduced HFD-induced obesity in mice. Moreover, preliminary findings from our groups have found that BAT histomorphology in mice on HFD with VSL#3 supplementation leads to restored BAT structure compared to mice fed HFD (unpublished data). Overall, these studies strongly indicate that the gut microbiome plays an important role in the regulation of BAT activity. Further studies that establish how these two organ systems are linked may allow for the development of novel therapeutics for the treatment of obesity and diabetes.

## 6. Browning in Humans

Until recently, it was widely believed that BAT was present in significant amounts only in human infants and regressed with age in adults [26]. However, FDG-PET/CT imaging has confirmed BAT activity in the supraclavicular regions in adult humans [3, 5] (Figure 1). In the original studies, BAT activity was inversely associated with body mass index and increased in response to cold exposure. Subsequent studies confirmed the identity of the tissue as BAT due to the presence of Ucp1^+^ cells and various other molecular markers consistent with BAT [3]. Since then, the scientific community has shown a great deal of interest in understanding BAT function in adults and its possible use as a therapeutic agent to counter obesity.

The BAT activity induced by cold exposure, diet, or pharmacological agents is correlated with increased energy expenditure. In some population studies, resting plasma glucose and lipid levels are inversely related to BAT activity [96]. Cold exposure increases glucose disposal specifically in BAT, and this response seems to be absent in obese individuals. Whole-body glucose disposal, glucose oxidation, and insulin sensitivity were enhanced in individuals with significant amounts of BAT, whereas these effects were blunted in obese individuals who did not have detectable BAT activity [97]. Together, these results support the notion that BAT serves an important role in modulating the risk of complications derived from obesity. Cold exposure is one of the more established environmental factors impacting variability in BAT activity. Short (10-day) cold exposure resulted in increased cold-induced glucose uptake in BAT, as assessed by FDG-PET. Cold-induced glucose uptake in BAT was positively related with glucose uptake in visceral WAT. Cold-induced skeletal muscle glucose uptake tended to increase upon cold acclimation, which was paralleled by increased basal GLUT4 localization in the sarcolemma, as assessed through muscle biopsies. These metabolic adaptations to prolonged exposure to mild cold may lead to improved glucose metabolism or prevent the development of obesity-associated insulin resistance and hyperglycemia [98].

Other environmental changes, such as increased exercise or injury, are known to modulate the relative activity of BAT. For instance, in certain populations, such as endurance athletes, chronic exercise is a common activity. The effect of exercise on the browning of adipose tissue is now fairly well-established and is consistent with the metabolic demands of exercise. BAT is, after all, a metabolically inefficient organ when thermogenesis is nonessential. For instance, Singhal et al. studied 24 women (16 athletes and 8 nonathletes) between 18 and 25 years of age. BAT volume and activity in athletes tended to be lower than in nonathletes. Moreover, BAT volume correlated inversely with lean mass and positively correlated with percent body fat, serum irisin, and serum thyroid hormones. This demonstrates that brown fat may undergo adaptive reductions in cases where there is a large energy deficit, such as in athletes with a chronic exercise routine [99].

Interestingly, surgical trauma can induce adipose tissue browning at the site of injury and, importantly, in the contralateral inguinal depot. However, merely an incision alone was not enough to produce browning, and browning was independent of surgery-associated body temperature changes and weight loss. Thus, adipose trauma as an unexpected driver of selected local and remote adipose tissue browning has important implications for the biologic response to surgical injury [100].

Obesity is associated with WAT inflammation and in some cases, localized fibrosis, impaired adaptive thermogenesis, and increased lipolysis. Prostaglandins (PGs) are powerful lipid mediators that have diverse hormone-like effects in many organs and tissues. Targeted LC-MS/MS lipidomic analysis of human omental WAT collected from obese patients undergoing bariatric surgery identified increased PGE_2_ levels in the face of COX-2 induction and unchanged expression of terminal prostaglandin E synthases. Importantly, exogenous addition of PGE_2_ to WAT explants significantly reduced the expression of fibrogenic genes and inhibited the expression of inflammatory genes (i.e., IL-6 and MCP-1). Moreover, PGE_2_ induced expression of Ucp1 and Prdm16 in WAT and adipocytes, but not preadipocytes, suggesting that PGE_2_ might induce the transdifferentiation of adipocytes toward beige cells. Finally, isoproterenol-induced adipocyte lipolysis was inhibited by PGE_2_. Collectively, these findings suggest that PGE_2_ is a critical regulator of inflammation, fibrosis, and impaired adaptive thermogenesis and lipolysis in human obese visceral WAT [101].

While the class of *β*3-adrenergic receptor (AR) agonists stimulates rodent BAT, this activity has never been demonstrated in humans. Cypess et al. demonstrated that treatment with mirabegron, a *β*3-AR agonist currently approved for overactive bladder, led to higher BAT metabolic activity as measured via FDG-PET-CT in healthy male subjects and further increased resting metabolic rate. This study suggests that *β*3-AR agonists have potential to stimulate human BAT thermogenesis and may be promising targets for the treatment of metabolic disease [102].

The role of BAT in lipid metabolism has not been explored as greatly as its role in thermogenesis and energy dissipation. Chondronikola et al. showed that in overweight/obese men exposed to prolonged, nonshivering, cold, and thermoneutral conditions, BAT volume was significantly associated with increased whole-body lipolysis, triglyceride-free fatty acid (FFA) cycling, FFA oxidation, and adipose tissue insulin sensitivity. Functional analysis of BAT and WAT demonstrated the greater thermogenic capacity of BAT compared to WAT, while molecular analysis revealed a cold-induced upregulation of genes involved in lipid metabolism only in BAT. The accelerated mobilization and oxidation of lipids upon BAT activation support a putative role for BAT in the regulation of lipid metabolism in humans [103].

The reported susceptibility of children exposed to gestational diabetes mellitus to metabolic dysfunction might involve epigenetic programming of impaired BAT function. Fetal exposure to maternal hyperglycemia is associated with DNA methylation variations in genes involved in BAT genesis and activation. Maternal glycemia during the second and third trimester of pregnancy is negatively correlated with placental DNA methylation levels of* PRDM16* and* BMP7* and positively correlated with placenta* Pgc1α* DNA methylation and cord blood leptin levels. Moreover, variations in* Prdm16* and* Pgc1α* DNA methylation levels are also correlated with cord blood leptin levels. These results suggest that maternal hyperglycemia during pregnancy is associated with altered placental DNA methylation patterns in BAT-related genes and that these epigenetic changes may link maternal glycemia and fetal adiposity [104].

## 7. Barriers to the Therapeutic Usage of Browning Agents

It is important to note that thermogenesis as a means of body weight regulation is not a novel concept and should still be viewed carefully with regard to safety. For instance, dinitrophenol, a mitochondrial uncoupling agent, was shown to be effective for weight loss, but patients treated with dinitrophenol presented with hyperthermia and other severe issues as side effects [105]. Also, thyroxine and catecholamines induce brown and beige adipocyte activity but the side effects of these compounds limit their use in obesity treatment. Furthermore, species variations in pharmacological responses may result in practical obstacles. For example, the *β*3-AR agonists CL 316243 and mirabegron potently activate brown and beige adipocytes and reduce adiposity in mice but the drug has no effect on adiposity in humans along with risk of hyperthermia.

A hypermetabolic response (HR) can be defined as a physiological state of increased metabolic activity and is characterized by an abnormal increase in basal metabolic rate. HR is accompanied by an increase in release of free fatty acids and glycerol from fat, excessive glucose production from the liver, and excess amino acid release from muscles. As a result of these chain of events, there is a significant increase in resting energy expenditure. While browning of adipose tissue implies a state of increased energy expenditure and a high state of energy turnover in tissues, our understanding of how browning relates to the hypermetabolic response is still in its infancy. HR in cases of serious injuries such as burns, trauma, and cancer are conditions where browning would be contraindicated. For example, in burn patients, browning induces cachexia which leads to severe weight loss, systemic inflammation, and muscle and adipose tissue wasting [106, 107]. Browning has also been implicated in the development and accelerated progression of atherosclerosis [108] and hepatic steatosis [109], possibly due to excessive lipolysis in hyperactive BAT. Thus, going forward to consider the potential for deleterious side effects and/or unintended metabolic consequences inherent in some browning regimes will be important.

## 8. Summary and Conclusion

The epidemic of obesity and diabetes presents significant global health concerns and supports further investigation into the treatment and prevention of metabolic disorders. The thermogenic capacity of BAT and its avidity for glucose makes it a plausible therapeutic target for inducing weight loss or improving blood sugar control. The crucial objective now is to identify molecules or compounds which will selectively act on adipocytes and provide the intended metabolic benefits of enhanced fat oxidation, reduced body fat, and improved glucose homeostasis, with minimal side effects. Currently, some of the most promising molecular targets for promoting BAT thermogenesis include BMP7, BMP8b, Cox-2, and Fgf-21, but their overall actions in other facets of metabolism and physiology require further investigation. The literature has also consistently shown that reduced ambient temperature induces BAT activity; however practical implementation of cold therapy is a logistical issue that also requires future work. Despite the remarkable progress over the last decade in unraveling the biology of BAT, much remains to be done in order to translate basic findings to clinically significant treatments for metabolic disease.

## Figures and Tables

**Table 1:** Several browning agents (genes, microRNAs, and pharmacological, chemical, or plant-based products) that induce browning/browning in adipose tissue and provide protection from HFD-induced obesity.

Browning agents	Effect on browning and metabolic outcome	Reference
Gain of function/overexpression		
Ucp1	Less lipid accumulation in adipocytes, resistance to HFD-induced obesity, beige cell phenotype in WAT, atrophy of BAT, and reduced Ucp1 and mitochondrial DNA content in BAT	[38, 39]
Prdm16	Beige adipocyte induction in WAT when stimulated by b-AR, increased energy expenditure, limited weight gain, and improved glucose tolerance in response to a HFD	[40]
Cox2	Ucp1 induction in WAT via stimulation of *β*-AR, increased thermogenesis, attenuated weight loss and energy expenditure, and protection against HFD-induced obesity	[44, 45]
LXA4	Higher LXA4 levels were related to browning of WAT, leaner body type, increased energy expenditure, and increased thermogenesis	[46, 47]
Foxc2	Increased b-AR-cAMP-PKA signaling was associated with reduced fat mass and browning of WAT and protection against HFD-induced obesity	[48]
Pten	BAT in Pten-overexpressing mice had high levels of Ucp1 and increased energy expenditure	[49]
AM2	Overexpression in adipose tissue led to reduced acetylation of Pgc1*α* and favorable interaction between Pgc1*α*, Prdm16, and Ucp1 in adipocytes, induction of browning in WAT, and protection against HFD-induced obesity	[52]

Loss of function/knockdown		
BMP7	Absence of BMP7 led to reduced interscapular BAT at birth	[53, 54]
Smad3/Tgfb	Improved glucose homeostasis with induction of beige cells in WAT which provided protection against obesity and increased mitochondrial bioenergetic profile of WAT	[56]
ABHD6	Increased energy expenditure, cold-induced thermogenesis, Ucp1 expression in WAT, fatty acid oxidation, browning of WAT, protection against HFD-induced obesity, and associated complications	[58]
Ga	Activation of G*α* signaling abrogates brown adipogenesis, whereas expression of G*α* signaling is inversely correlated with Ucp1 expression in WAT	
Folliculin	Mitochondrial uncoupling proteins as well as other markers of brown fat are upregulated in both white and brown FLCN-null adipose tissues	[51]
IEX-1	Induced browning of WAT, enhanced thermogenesis with markedly less weight gain, and increased energy expenditure on HFD	[59]
LXR*β*	Induction of beige adipocytes in WAT of mice fed a normal diet with improved metabolic phenotype	[60]

MicroRNAs and browning		
miR-455	Expressed in BAT-specific manner, induced by cold exposure, and induced browning in subcutaneous fat upon cold exposure	[64]
miR-92a	Inversely correlated with BAT activity in humans and could be used as a potential biomarker for BAT activity in mice and humans	[65]

Pharmacological and plant-based browning agents		
CL 316243	*β*3AR agonist induces browning in WAT, strong response to cold in the form of increased thermogenesis, increased Ucp1 mRNA in WAT and BAT upon treatment, weight loss, and improved energy expenditure	[66–69]
Gleevec	Increased browning of WAT and rate of energy expenditure, acting by blocking PPARg phosphorylation	[70]
Acetate	Increased heat production from brown and beige adipocytes corroborated by increased Ucp1 and Prdm16 expression in WAT	[71]
TZDs	Transforming WAT into BAT-like tissue with increased angiogenesis	[72]
PGE2	Browning induction in WAT with improved angiogenesis	[72]
Slit2 derived secretory product	Acting via Prdm16 to regulate beige adipocyte induction, increasing Ucp1 expression, and promoting adipose thermogenesis resulting in increased energy expenditure	[61]
Butyrate	Increased expression of Ucp-1 and Pgc1*α* and protected high fat diet-induced obesity in mice	[73]
Rapamycin	*β*AR-dependent increase in Ucp1 expression and expansion of beige adipocyte in WAT	[62]

Plant-based browning agents		
Butein	Ucp1 induction in WAT, mediated through Prdm4, leads to increased energy expenditure and stimulates generation of thermogenic adipocytes	[74]
Resveratrol	Reducing adipose tissue inflammation and increased expression of genes associated with the browning of adipose tissue	[75]
*β*-Lapachone	Stimulating the browning of WAT, increased expression of BAT-specific genes, decreased body weight gain, and ameliorated HFD effects	[76]
Capsaicin	Acting via TRPV1 channel, increased the expression of Pgc1*α* and Prdm16 and induced browning in adipose tissue, and provided protection from HFD-induced obesity	[77]
